# Immunohaematological reference values for HIV-negative healthy adults in Botswana

**DOI:** 10.4102/ajlm.v1i1.5

**Published:** 2011-12-15

**Authors:** Madisa Mine, Sikhulile Moyo, Penny Stevens, Kurt Michael, Vladimir Novitsky, Kgomotso Makhaola, Aida Asmelash, S’khatele Molefhabangwe, Elias Woldegabriel, Gaseboloke Mothowaeng, Talkmore Maruta, Charity Kamhukamwe, Phibeon M. Mangwendeza, Molly Holmes-Pretorius, Isaac Mtoni, Modisa Motswaledi, Rosemary Musonda, Ndwapi Ndwapi, Joseph Makhema, Richard Marlink, Khumo Seipone, Tendani Gaolathe, Max Essex

**Affiliations:** 1 National Health Laboratory, Gaborone, Botswana; 2 Botswana-Harvard AIDS Institute Partnership (BHP), Princess Marina Hospital, Gaborone, Botswana; 3 Safety Monitoring in International Laboratories (SMILE), Johns Hopkins University, Baltimore, Maryland, United States; 4 Department of Clinical Services, Ministry of Health, Gaborone, Botswana; 5 Department of HIV/AIDS Prevention and Care, Ministry of Health, Gaborone, Botswana; 6 Harvard School of Public Health, Boston, Massachusetts, United States

## Abstract

**Background:**

Clinical laboratories in Botswana have relied entirely on the reference intervals for normal immunohaematological values provided by manufacturers’ kits and textbooks.

**Objectives:**

The aim of this study was to determine the means, medians, 2.5th and 97.5th percentile reference intervals, for normal immunohaematological values in healthy adults in Botswana.

**Method:**

A total of 261 healthy participants comprising 126 men (48%) and 135 (52%) women were enrolled in the southern part of Botswana, and immunological and haematological laboratory parameters were measured.

**Results:**

The mean age was 28.8 (95% Confidence Interval [CI] 27.7–29.8) years, with a median of 27 years and a range 18–66 years. The mean haemoglobin level was significantly lower for women (12.4 g/dL; 95% CI 12.1% – 12.7%) than men (15.1 g/dL; 95% CI 14.9% – 15.3%). The women’s haemoglobin reference values (9.0 g/dL – 15.0 g/dL) levels were lower than observed in predominantly White populations (12.0 g/dL – 16.0 g/dL), but comparable with regional consensus reference intervals (9.5 g/dL – 15.8 g/dL) recently defined for East and Southern Africa.

**Conclusion:**

The established values provide an important tool for patient management and could influence decisions on inclusion of participants and adverse events in clinical trials conducted locally.

## Introduction

The human immunodeficiency virus (HIV) constitutes a major public health problem in Botswana with a prevalence of 17.6%^1^ in the general population and 31.8% amongst pregnant woman.^2,3^ Botswana is one of the countries in Africa that has led a very high response to the epidemic through a range of multilevel interventions, including the widespread access to antiretrovirals (ARVs),^4,5^ and has built capacity to conduct international prevention, treatment and vaccine trials.^6^ A valid scientific safety evaluation of ARVs and HIV vaccines relies on the availability of locally defined reference values for parameters of clinical interest.^7^

Reference intervals are essential for monitoring patho-physiological changes after infection or disease states, or following the administration of drugs in therapeutic or clinical interventions and vaccine studies. Several multicenter trials, including Phase I and/or Phase II vaccine studies and global use of ARVs, have increased the need for regional and locally established reference values.^8^ Reference values vary considerably in different populations, geographical regions, climate, race and dietary habits.^9,10,11,12,13,14,15^ Reference intervals studies conducted in African countries have shown differences within populations and sometimes within sub-groups, and marked differences with values from White populations.^8,13,16,17,18^ These studies have also observed significant differences with values from developed countries, in haematologic parameters such as haemoglobin and absolute neutrophil, which are normally used for toxicity grading in clinical trials, and consequently have implications in patient management and the conduct of clinical trials.^18,19,20,21^ A significant number of potential African volunteers are excluded from participating in vaccine trials because screening, enrollment and follow-up monitoring is mostly based on values derived from predominantly White populations. A study in Uganda^19^ documented an exclusion rate of as high as 69% of vaccine clinical trial participants because of haematologic abnormalities, 83% of whom could have been included in the studies if local reference ranges were used. Kibiya et al.^8^ observed that the lower limit of neutrophil counts and haemoglobin reference ranges qualify to be graded as moderate and severe adverse events, respectively, according to the Division of AIDS (DAIDS) toxicity grading table.^22^

Determination of the CD4+ T-lymphocyte counts play a pivotal role on the initiation and monitoring of patients on highly active antiretroviral therapy (HAART); hence the urgent need for establishing CD4+ lymphocytes reference intervals for local populations. Some studies have observed that Black people and Afro–Caribbean people have lower total white cell counts, and neutrophil and platelet counts, than White people.^9,23,24^ Immunohaematological reference values for a healthy Botswanan population have never been established formally, whereas in many African countries, it is common practice to use reference values established in predominantly White populations, which may not be representative of the Botswanan population (which comprises mostly Black people). In order to conduct investigation drug (IND) and vaccine trials successfully, an assessment of the local reference intervals is required.^25,26,27^ The aim of this study was to establish the immunohaematological reference values in healthy adults in Botswana following the Clinical Laboratory Standards Institute (CLSI) (formerly NCCLS) guidelines^28^ in order to improve patient care, the participation of individuals in clinical trials and the evaluation of adverse events.

## Materials and methods

### Study design, setting and population

A cross sectional study was conducted amongst healthy adult volunteers that were recruited at the voluntary counselling and testing center (VCT) known as *Tebelopele* (a Setswana name that means ‘foresight’*)* from around Gaborone, the capital city of Botswana. Potential volunteers who tested HIV-negative in a parallel testing algorithm that used Determine HIV-1/2 (Abbott Laboratories, IL, USA) and UniGold HIV (Trinity Biotech, Ireland) rapid test kits, were recruited into the study. The rapid HIV results were confirmed in the laboratory by using two enzyme-linked immunosorbent assays as per national guidelines.

### Study procedures

Potential apparently healthy HIV-negative volunteers who consented to participate in the study were included. A brief screening questionnaire was administered by experienced study nurses. The questionnaire was designed using the Clinical Laboratory Standards Institute (CLSI) guidelines^28^ (formerly NCCLS) and the following categories were excluded:

- pregnant
- breastfeeding
- patients who had been inpatients in a hospital or who had been subjectively ill during the last month
- patients receiving medical treatment
- patients who had a recent or recurrent infection, including HIV and malaria
- patients who had smoked in the hour before blood was drawn
- patients who had donated blood in the preceding month.

Participants had access to their HIV-testing results through voluntary counselling and testing protocol, and obtained all other results through a registered physician for review and referral to appropriate care. Participants with underlying clinical conditions were excluded from the analysis as well.

## Laboratory procedures

### Blood collection and HIV serology

Approximately 15 mL whole blood was collected from the cubital vein with a vacutainer system in K_3_EDTA. Samples were analysed on the day of collection. HIV status was further confirmed in plasma from the samples by using an enzyme-linked immunosorbent assays Vironostika HIV Uniform II plus O (BioMérieux France, Marcy l’Etoile, France) and Murex HIV-1.2.O Murex (Biotech, Dartford, UK) according to manufacturer’s instructions. A parallel algorithm was applied and the tests were performed at the Botswana Harvard HIV Reference Laboratory, following national guidelines that included confirmation with Western Blot or DNA PCR for all discordant results.

### Flow cytometric analysis

T-lymphocyte subsets were analysed on a FACSCalibur flow cytometer (Becton Dickinson Immunocytometry Systems, San Jose, California) by using a four-colour immunofluorescence reagent (BD MultiTEST CD3 fluorescein isothiocyanate [FITC]/CD8, phycoerythrin [PE]/CD45, peridinin chlorophyll protein [PerCP]/CD4, allophycocyanin [APC]), and TruCOUNT tubes according to the manufacturer’s instructions. The list-mode data was acquired and analysed with MultiSET software (Becton Dickinson Immunocytometry Systems). The instrument used was validated prior to use and it was monitored daily with TRUcount controls materials; it was also enrolled in an external quality assurance scheme by the United Kingdom External Quality Assessment Scheme (UKNEQAS). Analysis was conducted by trained and competent personnel.

### Haematological analysis

Haematology parameters were determined from whole blood by using the Sysmex XE-2100 (Sysmex, Kobe, Japan), according to the manufacturer’s instructions. The XE-2100 is capable of measuring 32 parameters, including the white blood cell (WBC) 5-part differential into lymphocytes, monocytes, eosinophils, neutrophils and basophils. It also provides a 14-parameter haemogram, as well as an integral reticulocyte analysis that includes an immature reticulocyte fraction, a nucleated red blood cell (NRBC) count and calculated parameters. These calculated parameters are, the mean corpuscular volume (MCV) (fL), the corpuscular haemoglobin (MCH) (pg), the mean corpuscular haemoglobin concentration (MCHC) (g/dL), and the RBC distribution width by standard deviation (RDW-SD) (fL). The accuracy and precision of the instrument was monitored daily by using commercial quality control materials, as well as quarterly through enrollment in an external quality assurance scheme by the College of American Pathologists (CAP). Trained and competent personnel performed the analysis.

## Ethical considerations

The study was approved by the Health Research and Development Committee (HRDC) of the Ministry of Health in Botswana. Written informed consent was obtained from participants prior to their enrollment. Those people found to be HIV-positive were excluded from the study, and referred for HIV care and treatment clinics for further management. CD4 and haematology results that were obtained, were offered free of charge as per the Botswanan guidelines.

### Statistical analysis

Reference intervals were calculated according to the CLSI guidelines document C28-A2 ^28^ by applying non-parametric methods. The medians were calculated and reference values were determined as the 2.5th and 97.5th percentiles, respectively, of the distribution of reference values. The mean, median, and standard deviation values were calculated for each immunohaematological parameter. We used confidence ratio, which is the ratio of (average confidence interval width) to (reference interval), as described by Rhoads,^29^ to evaluate the impact of the sample size. CLSI recommends a minimum of 120 healthy patients per group. The non-parametric Mann-Whitney U test was used to determine any statistically differences between laboratory values for men and women. *P*-values < 0.05 were considered significant. All statistical analyses were carried out with EP Evaluator Release 8 (David G. Rhoads and Associates, Kennett Square, Pennsylvania, USA) and Stata 11.0 (SataCorp, College Park, Texas, USA).

## Results

Screening and enrollment started in May 2008 and ended in June 2008. A total of 294 individuals were screened: 145 men (49%) and 149 women (51%). A total of 33 (11.2%) of screened volunteers were excluded, following enrollment based on defined exclusion criteria. The remaining 261 participants’ samples were included in the study, and comprised 135 women (51%) and 126 men (49%). The mean age was 28.8 (95% CI, 27.7–29.8) years, with a median of 27 and a range 18–66 years.

In our results (Table 1 and Table 2) we show the means, medians and 95th percentile reference values, according to gender, for CD4+ and CD8+ T-lymphocytes absolute values and percentages. The median CD4 cell count in the women (924 cells/mm^3^) was significant higher than in the men (744 cells/mm^3^), with *p* < 0.05. The calculated combined reference intervals for CD4+ and CD8+ T-lymphocytes were 261 cells/mm^3^ – 1667 cells/mm^3^ and 261 cells/mm^3^ – 1538 cells/mm^3^ for men, and 268 cells/mm^3^ – 1667 cells/mm^3^ for women (Table 1). The reference intervals for CD8+ T-lymphocytes (Table 2) were comparable to those obtained from a blood donor population. The study furthermore shows that the CD4% and CD8% reference intervals for women were 27–63, for men 27–60, and for both sexes 27–63 (Table 3). The CD8% reference intervals were 12–46 for women, 11–45 for men, and 11–46 for both sexes. The absolute CD4 and CD8 values for Botswana were lower than for most African countries (Table 4).

**TABLE 1:** CD4+ cell counts for healthy HIV-negative adults in Botswana.

Source	Sex	*N*	CD4+ cell counts (cells/mm^3^)					
			Mean	s.d.	95% CI	Median	Range	2.5th–97.5th percentile
General population	Combined	261	859	284	824–894	827	261–1667	374–1527
	Female	135	949	288	900–998	924^*^	268–1667	467–1603
	Male	126	762	247	719–805	744^*^	261–1538	333–1275
Blood donors	Combined	437	759	245	736–782	726	171–1652	366–1318
	Female	143	827	245	787–868	786	344–1558	438–1328
	Male	294	725	238	698–753	698	171–1652	366–1252

*Source*: Blood donor data quoted from Bussmann H, Wester CW, Masupu KV, et al. Low CD4+ T-lymphocyte values in HIV-negative adults in Botswana. Clin Diagn Lab Immunol. 2004;11:930–935. PMid:15358655, PMCid:515279

*N*, Number of subjects; CI, confidence interval; s.d., standard deviation.

* *p* < 0.001 (Mann-Whitney U test used to calculate *p*-values).

**TABLE 2:** CD8+ cells counts for healthy HIV-negative adults in Botswana.

Source	Sex	*N*	CD8+ cell counts (cells/mm^3^)					
			Mean	s.d.	95% CI	Median	Range	2.5th–97.5th percentile
General population	Combined	261	540	210	514–566	522	173–1166	225–1053
	Female	135	557	211	521–593	546^*^	184–1093	234–1057
	Male	126	522	208	486–558	497^*^	173–1166	180–1058
Blood donors	Combined	437	509	205	490–528	488	90–1573	190–1014
	Female	143	523	203	490–557	494	155–1198	228–1062
	Male	294	502	205	479–526	485	90–1573	178–994

*Source*: Blood donor data quoted from Bussmann H, Wester CW, Masupu KV, et al. Low CD4+ T-lymphocyte values in HIV-negative adults in Botswana. Clin Diagn Lab Immunol. 2004;11:930–935. PMid:15358655, PMCid:515279

*N*, Number of subjects; CI, confidence interval; s.d., standard deviation.

* *p* < 0.001 (Mann-Whitney U test used to calculate *p*-values).

**TABLE 3:** CD4% and CD8% cells counts for healthy HIV-negative adults in Botswana.

Cell type	Sex	*N*	Cell counts (cells/mm^3^)					
			Mean	s.d.	95% CI	Median	Range	2.5th–97.5th percentile
CD4	Combined	261	43	6.7	42–44	43	27–63	29–57
	Female	135	45	6.3	44–46	45^*^	27–63	32–57
	Male	126	41	6.8	40–42	41^*^	27–60	28–34
CD8	Combined	261	27	6.6	26–28	27	11–46	16–41
	Female	135	26	6.1	25–27	26^**^	12–46	16–40
	Male	126	28	7	27–29	29^**^	11–45	16–43

*N*, Number of subjects; CI, confidence interval; s.d., standard deviation.

* *p* < 0.001

** *p* = 0.011 (Mann-Whitney U test used to calculate *p*-values).

**TABLE 4:** Comparison of ratio of CD4+ to CD8+ and absolute CD4+, CD8+ and lymphocyte counts between different populations.

Source population	Sample size (*n*)	Reference	Absolute CD4+		Absolute CD8+		Absolute Lymphocyte Count		CD4 / CD8 T-cell ratio	
			Mean value	Reference intervals	Mean value	Reference intervals	Mean value	Reference intervals	Mean value	Reference intervals
Botswanan	260	Current study	859	261–1667	540	173–1166	-	-	1.65	0.79–2.88
Botswanan (Blood donors)	437	23	759	171–1652	509	90–1573	-	-	1.63	0.40–55.83
Eastern and Southern African	2105	18	-	457–1628	-	230–1178	-	1200–3700	-	-
Ethiopian	142	16	775	366–1235	747	311–1618	1857	1032–3432	1.20	0.40–2.40
Ugandan	183	13	1256	559-2333	668	253–1396	2666	1453–4448	2.16	0.68–4.40
US-based Comparison interval	-	30	30	518–1981	-	270–1335	-	1000–4800	-	-

Note: Please see the full reference list of the article, Mine M, Moyo S, Stevens P, et al. Immunohaematological reference values for HIV-negative healthy adults in Botswana. Afr J Lab Med. 2011;1(1), Art. #5, 7 pages. http://dx.doi.org/10.4102/ajlm.v1i1.5, for more information.

Our research shows the reference intervals for the haematological parameters (mean; mean s.d; and 95% CI for mean, median, range 2.5th to 97.5th percentile) partitioned by gender (Table 5 and Table 6). The red blood cell (RBC) parameters (median haemoglobin, haematocrit, and RBC) and WBC were statistically different according to gender (*p* < 0.05). There were; however, no gender-specific differences observed for some white blood cell (WBC) subsets (neutrophils, eosinophils, lymphocytes, reticulocytes and basophils), except for monocytes (*p* = 0.0089). Our research also shows haematological reference intervals from this study and those from certain African countries^14,16,18,19^ and non-African populations (Table 7).^30^ The haemoglobin reference intervals were generally higher than in East Africa, but lower than those from Ethiopia and US-based comparison populations.

**TABLE 5:** Means, medians and 95th percentile reference intervals for haematological parameters for 261 HIV-negative adults in Botswana.

Subject group	Parameter	WBC count (10^9^/L)	RBC count (10^9^/L)	Haemoglobin (g/dL)	Haematocrit (%)	Platelet count (10^9^/L)	Mean Cellular Volume (fL)	Mean Cell Haemoglobin (pg)	Mean Cell Haemoglobin Concentration (g/dL)
Male	Mean	5.1	5.20	15.12	43.38	277.80	83.68	29.25	34.9
	s.d.	1.54	0.39	0.99	2.59	60.00	4.03	1.84	1.03
	Median	4.84	5.18	15.3	43.3	277.5	84	29.5	35
	Reference range	2.9–7.9	4.20–6.30	11.90–17.10	36.10–49.30	141–494	73.80–95.60	23.40–33.30	31.80–37.60
Female	Mean	5.9	4.44	12.54	36.69	277.80	82.72	28.02	33.88
	s.d.	1.7	0.33	1.31	3.36	60.00	6.57	2.97	1.22
	Median	5.66	4.44	12.9	37.4	277.5	84.15	28.55	34.05
	Reference range	3.9–8.6	3.66–5.39	9.30–16.00	28.20–46.20	141–494	62.40–94.30	17.90–32.80	30.60–36.60
-	*p*-value^*^	< 0.05	< 0.05	< 0.05	< 0.05	< 0.05	0.8885	0.0017	< 0.05

RBC, red blood cell; WBC, white blood cell; s.d., standard deviation.

* *P*-values (Mann-Whitney U test) are for comparison of medians for male and female subjects.

**TABLE 6:** Means, medians and 95th percentile reference intervals of white blood cell subset percentages for 261 HIV-negative adults in Botswana.

Subject group	Parameter	Neutrophils	Lymphocytes	Monocytes	Eosinophils	Basophils	Reticulocytes
Male	Mean	52.13	36.63	8.04	2.69	0.44	10.11
	s.d.	8.98	9.08	2.17	2.02	0.26	3.40
	Median	52.4	37.6	7.9	2	0.4	9.2
	95% range	29.40–73.40	15.80–56.70	3.20–15.90	0.20–12.40	0.0–1.50	3.30–22.30
Female	Mean	52.19	37.69	7.74	2.36	0.44	9.45
	s.d.	10.04	8.76	2.15	2.02	0.25	3.39
	Median	52.85	8.42	7	1.8	0.4	9.3
	95% range	29.40–73.40	15.80–56.70	3.20–15.90	0.20–12.40	0.0–1.50	3.30–22.30
-	*p*-value	0.8076	0.3280	0.0089	0.2643	0.9334	0.2290

s.d., standard deviation.

* *P*-values (Mann-Whitney U test) are for comparison of medians for male and female subjects.

**TABLE 7:** Comparison of haematological parameters between different populations.

Parameter	Sex	Botswanan	South African	Kenyan	Ugandan blood donors	Eastern and Southern African	Ethiopian	US-based Comparison interval
Reference	-	(This Study)	33	18	13	18	16	30
WBC (10^9^/L)	Combined	3–10	-	2.8–8.2	2.8–8.2	3.1–9.1	3.0–9.8	4.5–11.0
	Male	-	3.92–10.4	-	-	-	-	-
	Female	-	3.90–12.6	-	-	-	-	-
RBC (10^12^/L)	Combined	-	-	-	-	-	-	-
	Male	4.4–6.0	4	.19–5.85	4.4–6.3	3.8–6.1	4.0–6.4	4.3–5.9	4.5–5.9
	Female	3.7–5.1	3.93–5.40	3.7–5.6	3.3–5.3	3.8–5.6	3.7–5.2	4.0–5.2
HGB (g/dL)	Combined	-	-	-	-	-	-	-
	Male	13–17	13.4–17.5	8.3–11.3	11.6–17.1	12.2–17.7	13.9–18.3	13.5–17.5
	Female	9–15	11.6–16.4	5.9–10.0	9.8–16.2	9.5–15.8	12.2–16.6	12.0–16.0
HCT (%)	Combined	-	-	-	-	-	-	-
	Male	38–49	39–51	40–50	33.8–49.5	35.0–50.8	41.6–55.1	41–53
	Female	29–43	34–48	30–50	28.3–46.8	29.4–45.4	35.3–48.8	36–46
PLT (10^9^/L)	Combined	160–395	-	120–411	109–384	126–438	-	150–350
	Male	-	171–338	-	-	-	97–324	-
	Female	-	186–454	-	-	-	98–352	-
MCH (pg)	Combined	-	-	-	-	-	-	-
	Male	24–33	27.8–34.8	23.3–33.8	23.0–33.8	-	-	-
	Female	20–32	26.1–33.5	21.3–33.0	24.8–32.7	-	-	-
MCHC (g/dL)	Combined	-	-	-	-	-	-	-
	Male	31–37	33.0–35.0	32.2–35.2	32.4–35.3	-	-	-
	Female	31–37	32.7–34.9	32.2–35.3	33–35.5	-	-	-
MCV (fL)	Combined	-	-	-	-	68-98	-	80–100
	Male	76–93	83.1–101.6	71.4–98.2	71–97	-	-	-
	Female	65–95	78.9–98.5	66.0–95.7	74–94.5	-	-	-
Neutrophils count (10^9^/L)	Combined	1.2–5.6	-	914–4715	0.9–3.9	1.0–5.3	-	1.8–7.7
	Male	-	1.6–6.98	-	-	-	-	-
	Female	-	1.6–8.3	-	-	-	-	-
Neutrophils (%)	Combined	33–69	-	40–60	22.2–59.3	25-66	-	40–70
	Male	-	32–76	-	-	-	-	-
	Female	-	34–72	-	-	-	-	-
Lymphocyte counts (10^9^/L)	Combined	1.0–3.6	-	1140–3454	1.2–3.7	1.2–3.7	0.96–3.47	1.0–4.8
	Male	-	1.4–4.2	-	-	-	-	-
	Female	-	1.4–4.5	-	-	-	-	-
Lymphocytes (%)	Combined	20–54	-	20–60	26.7–61.2	23-59	16–55.4	22–44
	Male	-	18–56	-	-	-	-	-
	Female	-	21–56	-	-	-	-	-
Monocyte counts (10^9^/L)	Combined	0.2–0.7	0.3–0.8	130–600	0.2–0.7	0.20–0.78	0.17–0.70	0–0.8
	Male	-	0.2–0.8	-	-	-	-	-
	Female	-	-	-	-	-	-	-
Monocyte (%)	Combined	4–13	-	3–11	4.7–12.7	4.5–13.1	4–10.7	4–11
	Male	-	4–12	-	-	-	-	-
	Female	-	3–10	-	-	-	-	-
Eosinophils count (10^9^/L)	Combined	0.01–0.50	0–0.95	30–1139	0.04–1.6	0.04–1.53	-	0–0.45
	Male	-	0–0.40	-	-	-	-	-
	Female	-	-	-	-	-	-	-
Eosinophils (%)	Combined	0–9	-	1–20	1.0–25	0.8–21.8	-	0–8
	Male	-	0–8	-	-	-	-	-
	Female	-	0–6	-	-	-	-	-
Basophils count (10^9^/L)	Combined	0.01–0.06	0-0.1	10–80	0.01–0.08	0.01–0.15	-	0–0.2
	Male	-	0–0.1	-	-	-	-	-
	Female	-	-	-	-	-	-	-
Basophils (%)	Combined	0–2	-	0–2	0.3–1.4	0.4–2.5	-	0–3
	Male	-	0–2	-	-	-	-	-
	Female	-	0–1	-	-	-	-	-

RBC, red blood cell; WBC, white blood cell; HGB, haemoglobin concentration; HCT, haematocrit; PLT, platelets; MCH, mean corpuscular haemoglobin weight ; MCHC, mean corpuscular haemoglobin concentration; MCV, mean corpuscular volume of single red cell.

Note: Please see the full reference list of the article, Mine M, Moyo S, Stevens P, et al. Immunohaematological reference values for HIV-negative healthy adults in Botswana. Afr J Lab Med. 2011;1(1), Art. #5, 7 pages. http://dx.doi.org/10.4102/ajlm.v1i1.5, for more information.

## Discussion

The reference intervals for immunohaematological and clinical chemistry parameters, which may serve as Botswanan standards for the interpretation of laboratory results, were established from 260 HIV-negative participants (134 women [52%] and 126 men [48%]) aged 18–66 years, from around Gaborone. As expected, the percentage CD4+ and CD8+ T-lymphocytes varied less between women and men. Malone et al.^31^ reported large fluctuations in repeated CD4+ cell counts in HIV-positive patients and can be explained, in part, by CD4+ cell count diurnal cycle, and also by high variability in total lymphocyte counts. HIV-negative women have a higher average CD4+ T-lymphocytes count than men, which confirms the findings of Bussmann et al.^32^ The CD4 cell counts are lower in Botswana than those observed in East Africa and USA. This has obvious implications on the clinical staging of AIDS and the assessment for disease progression.

The significant gender difference in red blood cell count, haemoglobin and haematocrit, agrees with other studies^16^ and with the well-established fact that men have higher values for the red blood cell parameters than women, partly because of the influence of the hormone androgen on erythropoiesis, and partly because of menstrual loss. The red blood cell parameters for the Botswanan population were within the intervals for the Ethiopian population, even though the expectation was that altitude induces erythropoiesis, which has accounted for the red blood cell parameters of Ethiopia that were consistently higher than those of many other African countries.^16^

The median haemoglobin level was significantly lower for women than for men, which is consistent with previous findings.^8,9,21,27^ The female haemoglobin reference value (9.0 g/dL to 15.0 g/dL) levels were lower than observed in predominantly White populations (12.0 g/dL to 16.0 g/dL), but comparable with East African and Southern African regional consensus reference intervals (9.5 g/dL to 15.8 g/dL) recently defined for East and Southern Africa. The Division of AIDS (DAIDS) has haematological criteria for grading the severity of potential vaccine-related adverse events.^22^ White blood cell and platelet counts, and haemoglobin levels are used as inclusion and/or exclusion criteria in many clinical trials. The lower limit of Botswana’s normal female reference range for haemoglobin qualifies as a moderate adverse event and a significant proportion of the woman may not qualify to be enrolled for Phase I and/or Phase II vaccine trials.

The methodologies in this study are commonly used, and should serve well as guidelines for the represented population. Laboratories that wish to adopt these reference intervals should explore any differences in methodology, and should verify the transference and appropriateness of the reference intervals to their laboratory.

### Limitations of the study

Although this study meets the minimum CLSI requirements for establishing valid reference intervals, it is be important to explore further geographic and ethic differences that may exist within the Botswanan population. One limitation of our study is that, despite our efforts to include only healthy subjects, the use of a questionnaire, as well as HIV screening and a review by a physician, it was not feasible to screen for all medical conditions that might have influenced the laboratory results. For instance, participants were not examined for other infections such as respiratory infections, recent transient gastroenteritis and sexually transmitted infections. All participants were adults drawn from voluntary counselling and testing facilities. When reporting reference values, the actual representativeness of the data is an important concern for the underlying population. As in most studies, we cannot totally exclude the possibility that there is some sampling bias because of use of a self-selecting population. Our analysis excluded participants who showed any signs or symptoms of disease, however, and it is thus likely that our data are representative of healthy adults from the southern region of Botswana. Gaborone is the capital city of Botswana and has a diversity of ethnic groups; consequently, reference values from this region are not likely to make a significant clinical difference. Because of access to a reference laboratory working under ISO and GCLP standards, it was not easy to include regions that are further away from the capital city, but this data is still considered to be a very important baseline for similar future studies. The data are also immediately applicable in studies and clinical care that is an improvement over the current practice of using reference values derived from elsewhere.

## Conclusion

This is the first study in Botswana to document haematological reference intervals for healthy adults. Our study shows that clinical reference values developed within the region are more appropriate for the Botswanan population than those adopted from developed countries. The established values provide an important tool for patient management and could influence decisions on the inclusion of participants, as well as the management of adverse events. It could also improve scientific validity in clinical trials that are conducted locally and regionally.
